# Deletion of *FoxN1* in the Thymic Medullary Epithelium Reduces Peripheral T Cell Responses to Infection and Mimics Changes of Aging

**DOI:** 10.1371/journal.pone.0034681

**Published:** 2012-04-13

**Authors:** Jianfei Guo, Yan Feng, Peter Barnes, Fang-Fang Huang, Steven Idell, Dong-Ming Su, Homayoun Shams

**Affiliations:** Health Science Center, The University of Texas, Tyler, Texas, United States of America; UC Irvine Medical Center, United States of America

## Abstract

Aging increases susceptibility to infection, in part because thymic involution culminates in reduced naïve T-lymphocyte output. Thymic epithelial cells (TECs) are critical to ensure normal maturation of thymocytes and production of peripheral T cells. The forkhead-class transcription factor, encoded by *FoxN1*, regulates development, differentiation, and function of TECs, both in the prenatal and postnatal thymus. We recently showed that expression of *FoxN1*, by keratin 14 (K14)-expressing epithelial cells is essential for maintenance of thymic medullary architecture, and deletion of *FoxN1* in K14 promoter-driven TECs inhibited development of mature TECs and reduced the number of total thymocytes. These findings are reminiscent of changes observed during normal thymic aging. In the current report, we compared the effects of K14-driven *FoxN1* deletion on peripheral T cell function in response to influenza virus infection with those associated with normal aging in a mouse model. *FoxN1*-deleted mice had reduced numbers of peripheral CD62L+CD44− naïve T-cells. In addition, during influenza infection, these animals had reduced antigen-specific CD8+ T-cell and IgG responses to influenza virus, combined with increased lung injury, weight loss and mortality. These findings paralleled those observed in aged wild type mice, providing the first evidence that K14-mediated *FoxN1* deletion causes changes in T-cell function that mimic those in aging during an immune response to challenge with an infectious agent.

## Introduction

The thymus is capable of generating T cells throughout life and is crucial for development, selection, and maintenance of peripheral T-cells. Aging reduces adaptive immunity to pathogens and lowers immune responses to vaccines, in part because thymic involution results in striking loss of progenitors, epithelial cells, and differentiating thymocytes, culminating in reduced naïve T-lymphocyte output [1]–[3].

In the mature thymus, a three-dimensional network of thymic epithelial cells (TEC) is a critical component of the thymic microenvironment that fosters the development of common lymphoid progenitors, which continuously migrate from the bone marrow to the thymus, and the maturation of early thymocyte progenitors to peripheral T lymphocytes. We have recently shown that expression of *FoxN1*, by keratin 14 (K14)-expressing epithelial cells is essential for morphogenesis and maintenance of the thymic medullary architecture, and deletion of *FoxN1* in K14 promoter-driven TECs (defined as “*FoxN1 K14*KO”) inhibited development of mature TECs and reduced the number of total thymocytes, although the proportions of CD4+, CD8+ and CD4+CD8+ cells were normal [4]. These findings in mice with K14-driven *FoxN1* deletion are reminiscent of changes observed during normal aging of the immune system.

In the current report, we compared the effects of K14-driven *FoxN1* deletion on peripheral T cell function with those associated with normal aging, focusing on a physiologically relevant model of influenza infection. We compared the peripheral T cell responses in aged wild type (WT) mice, K14-driven *FoxN1*-deleted mice and young WT mice.

## Results

### Loss of *FoxN1* and aging reduce the numbers of naïve T cells

We previously generated a *FoxN1* K14KO mouse, in which there was progressive prenatal and postnatal loss of *FoxN1* in K14+ medullary thymic epithelial cells (TECs), resulting in reduced thymic size and numbers of thymocytes [4]. These findings are similar to those in WT aged mice, suggesting that FoxN1 in K14+ TECs may contribute to aging-associated degeneration of the thymus. To test this hypothesis, we compared the phenotype and proliferative capacity of peripheral T cells in young WT mice, young *FoxN1* K14KO mice and aged WT mice.

**Figure 1:**
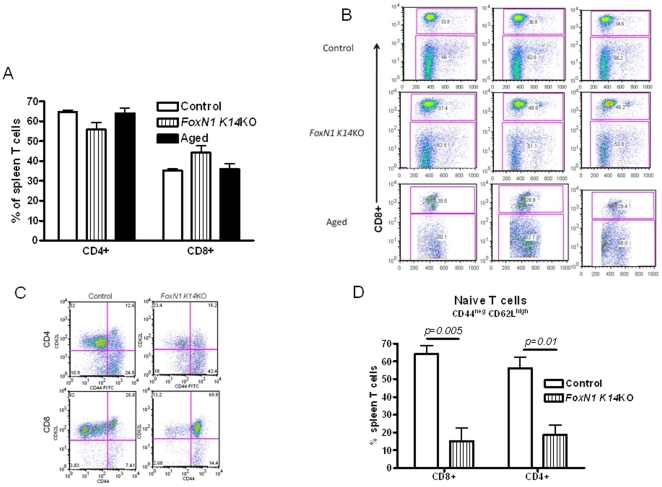
Analyses of T cell subsets of *FoxN1 K14KO*, aged and young control mice. (A) Spleen T cells from *FoxN1 K14KO*, aged and young control mice were stained with antibodies to CD3 and CD8. We gated on CD3+CD8+ cells to identify CD8+ T cells. CD3+CD8− T cells were considered CD4+ T cells (n = 3–5 per group). (B) Dot plot of three individual mice from panel A are shown for each group. (C and D) Spleen cells from *FoxN1 K14KO* and young control mice were stained with antibodies to CD3, CD8, CD4, CD62L and CD44. We gated either on CD3+CD4+ or CD3+CD8+ T cells. Representative histograms are shown in panel C. Means and SEMs of 3 mice/group are shown in panel D (p = 0.01 for CD4+ and p = 0.005 for CD8+ naïve T cells, comparing *FoxN1 K14KO* and young mice). Results are representative of 2 experiments.

We first measured the distribution of CD4+ and CD8+ T cells in the spleens of aged WT, young *FoxN1 K14*KO and young control WT mice. The proportions of CD4+ and CD8+ T cells in aged WT and young *FoxN1 K14*KO mice were similar to those in young WT mice (Figures 1A and B).

Next, we evaluated the distribution of naïve and memory T-cells, using the CD62L and CD44 markers. Naïve cells are CD62L+CD44−, and aging is associated with a marked reduction in the percentage of naïve T cells, with a corresponding restriction in the T cell repertoire [5]. We found that aged WT and *FoxN1 K14*KO mice had similar and marked reductions in the percentages of CD62L+CD44− naïve T cells, compared to findings in young WT mice, both in CD4+ and CD8+ subpopulations (Figures 1C and D).

**Figure 2:**
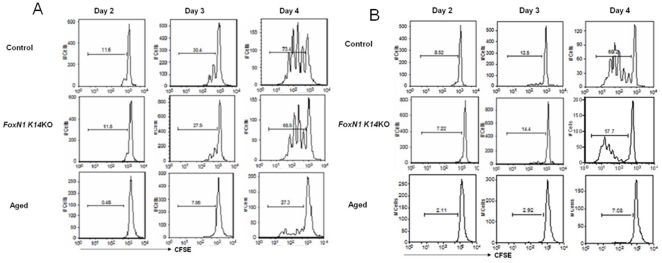
Poor peripheral T cell proliferation defect in aged mice. CD4+ or CD8+ T cells from naïve *FoxN1 K14KO*, aged and young control mice were isolated from spleens by positive immunomagnetic selection, stained with CFSE and stimulated with suboptimal concentrations of anti-CD3 and anti-CD8. Two, 3 and 4 days after stimulation, proliferation of CD4+ (A) and CD8+ (B) T cells were measured by flow cytometry. Three mice/group were used in two independent experiments.

Aged mice have poor peripheral T cell proliferation [6], [7]. To determine if *FoxN1 K14*KO mice shared this defect, we isolated spleen T cells, labeled them with CFSE and stimulated them in vitro with suboptimal concentrations of anti-CD3 and anti-CD28. CD4+ and CD8+ T cells from aged mice showed reduced proliferation, while cells from young *FoxN1 K14*KO mice had comparable proliferation to those of young WT mice (Figures 2A and B, respectively).

### Loss of *FoxN1* and aging reduce antigen-specific CD8+ T-cell and IgG responses in influenza infection

**Figure 3:**
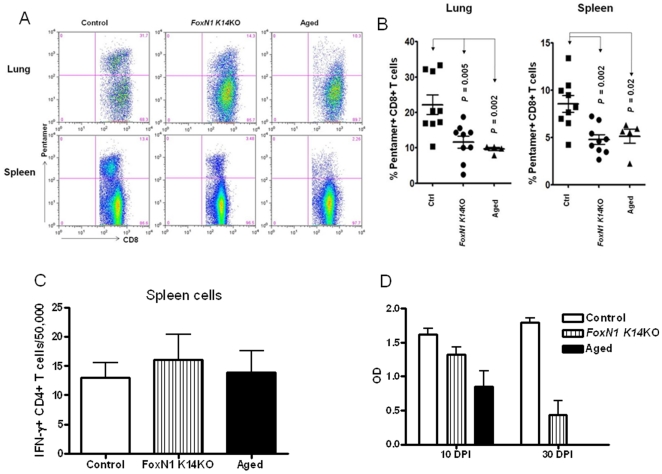
Role of *FoxN1* and aging on antigen-specific CD8+ and CD4+ T-cell and IgG responses to influenza infection. *FoxN1 K14KO*, aged and young control mice were infected with a sublethal dose of the influenza A virus PR8. (A and B) The frequency of antigen-specific CD8+ T cells in the lungs and spleens were measured 10 days later. MHC class I H2-Db pentamers, conjugated to R-phycoerythrin (R-PE) and loaded with the influenza virus NP_366–374_ (ASNENMETM) peptide, were used to detect antigen specific CD8+ T cells in lungs and spleens by flow cytometry. A representative histogram is shown in (A) and means and SEMs of 5–9 mice/group are shown in (B). (C) The frequencies of PR8-specific IFNγ-producing CD4+ T cells were measured by ELISPOT. Spleen CD4+ T cells were isolated by immunomagnetic selection from individual mice, 10 days after infection, then incubated with APCs on ELISPOT plates, pre-coated with anti-IFN-γ. APCs were splenocytes from naïve mice that were infected with IAV or with medium alone as controls, and irradiated. Plates were developed 24 hrs later and the frequency of IAV-specific IFN-γ producing cells was determined. (D) Anti H1 IgG levels in aged, *FoxN1 K14*KO and young control mice. Mice were infected with a sublethal dose of PR8 virus intranasally and levels of anti-H1 IgG were measured by ELISA. Some aged mice died by 30 days post-infection, so results are not shown for aged mice at this point. Means and SEMs of 5 mice/group are shown.

Protective immunity in influenza hinges on robust T and B cell responses, and the immune response to influenza is impaired in the elderly, resulting in increased morbidity and mortality [8], [9]. Hence, we used influenza infection as a model to determine if progressive loss of *FoxN1* mimicked the antigen-specific T- and B-cell responses of aging. Mice were infected with a sublethal dose of the influenza A virus PR8. Ten days later, the frequencies of antigen-specific CD8+ T cells in the lungs and spleens were measured by flow cytometry, using MHC class I H2-Db pentamers, conjugated to R-phycoerythrin (R-PE) and loaded with the influenza virus NP_366–374_ (ASNENMETM) peptide. As shown in Figures 3A and B, the numbers of influenza-specific CD8+ T cells in the lung and spleen were reduced by approximately 50% in young *FoxN1 K14*KO and aged WT mice, compared to young WT mice (*p*<0.05). However, the frequency of PR8-specific IFNγ-producing CD4+ T cells was similar in all three groups (Figure 3C). Because CD8+ T cells play a dominant role in clearing influenza virus [10], these results are consistent with a significant defect in the response to influenza in aged mice, which is replicated in the *FoxN1 K14*KO mice.

Humoral immunity also mediates protection against influenza, and the antibody response to influenza vaccination is reduced in the elderly [9]. To evaluate the antibody response in *FoxN1 K14*KO mice, we measured IgG to the H1 component of influenza A in the sera of PR8-infected mice, 10 and 30 days after infection. H1-specific IgG levels in aged mice were markedly reduced at 10 days after infection, and H1 IgG levels were significantly lower in young *FoxN1 K14*KO mice, although they were higher than those in aged mice (Figure 3D). By 30 days after influenza infection, some aged mice died, so results are only presented for young control and *FoxN1 K14*KO mice. At this time point, H1 IgG levels were markedly reduced in *FoxN1 K14*KO mice (Figure 3D), to a greater degree than at 10 days after infection. This suggests that *FoxN1 K14*KO mice have a reduced humoral response to influenza infection, similar to findings in aged WT mice.

Unlike the case for H1 IgG, IgA levels in the lung homogenates of *FoxN1 K14*KO mice and young WT mice were not significantly different, 4 and 10 days after influenza infection (data not shown).

### *FoxN1 K14*KO mice and aged mice have greater weight loss and lung injury from influenza virus infection

**Figure 4:**
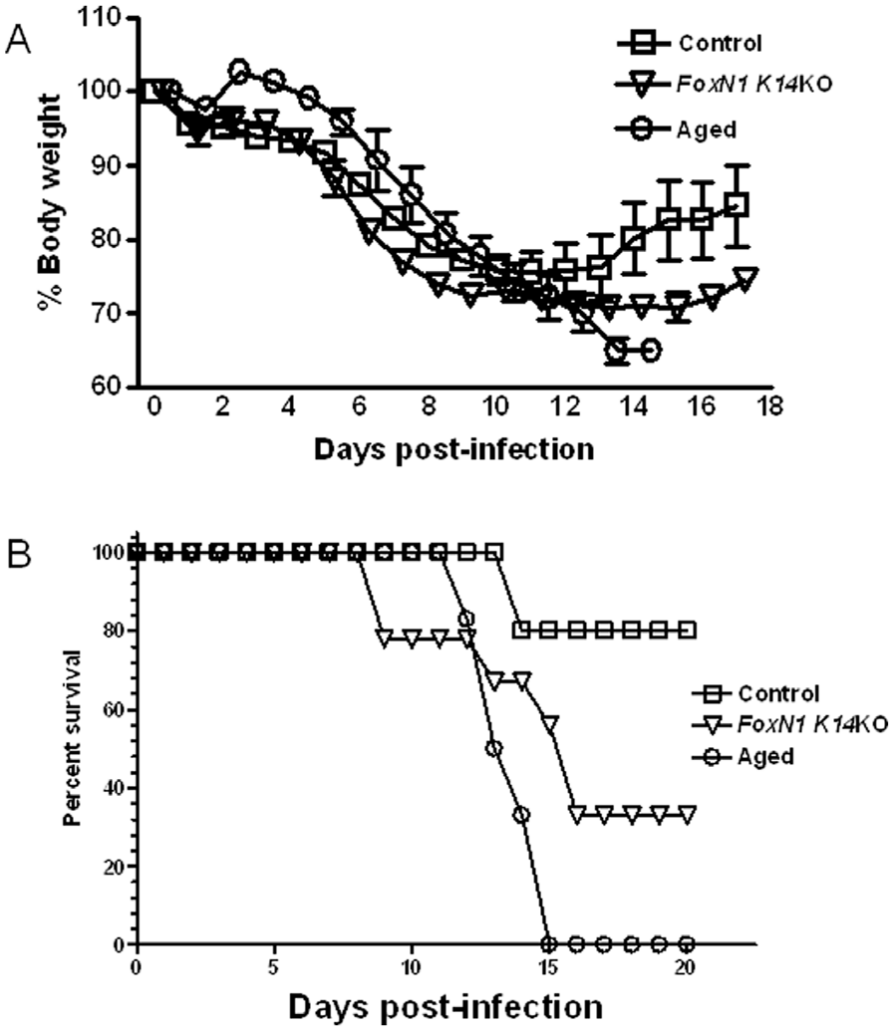
Weight loss and mortality among aged, *FoxN1 K14*KO and young control mice. *FoxN1 K14KO*, aged and young control mice were infected with 0.5 LD50 of PR8 virus and monitored daily. (A) Percent body weight was calculated, based on initial body weight at the time of infection. (B) Surviving mice were monitored for 8 weeks and there were no deaths after 16 days post-infection. The LD50 was calculated for young control mice. A representative of two experiments is shown.

Elderly persons have higher frequencies of pneumonia and death from influenza than young adults [9]. To determine if K14-mediated *FoxN1* deletion increases morbidity from influenza, we infected WT, *Foxn1 K14*KO and aged mice with 0.5 LD50 of influenza virus PR8, the LD50 being based on that for young control mice. All three groups of mice lost 20–25% of their body weight by 10–13 days after infection. However, young control WT mice began to regain weight after that point, whereas *FoxN1 K14*KO and aged mice did not (Figure 4A). In addition, all aged WT mice and 70% of *FoxN1 K14*KO mice died, compared to only 20% of young control mice (Fig. 4B).

**Figure 5:**
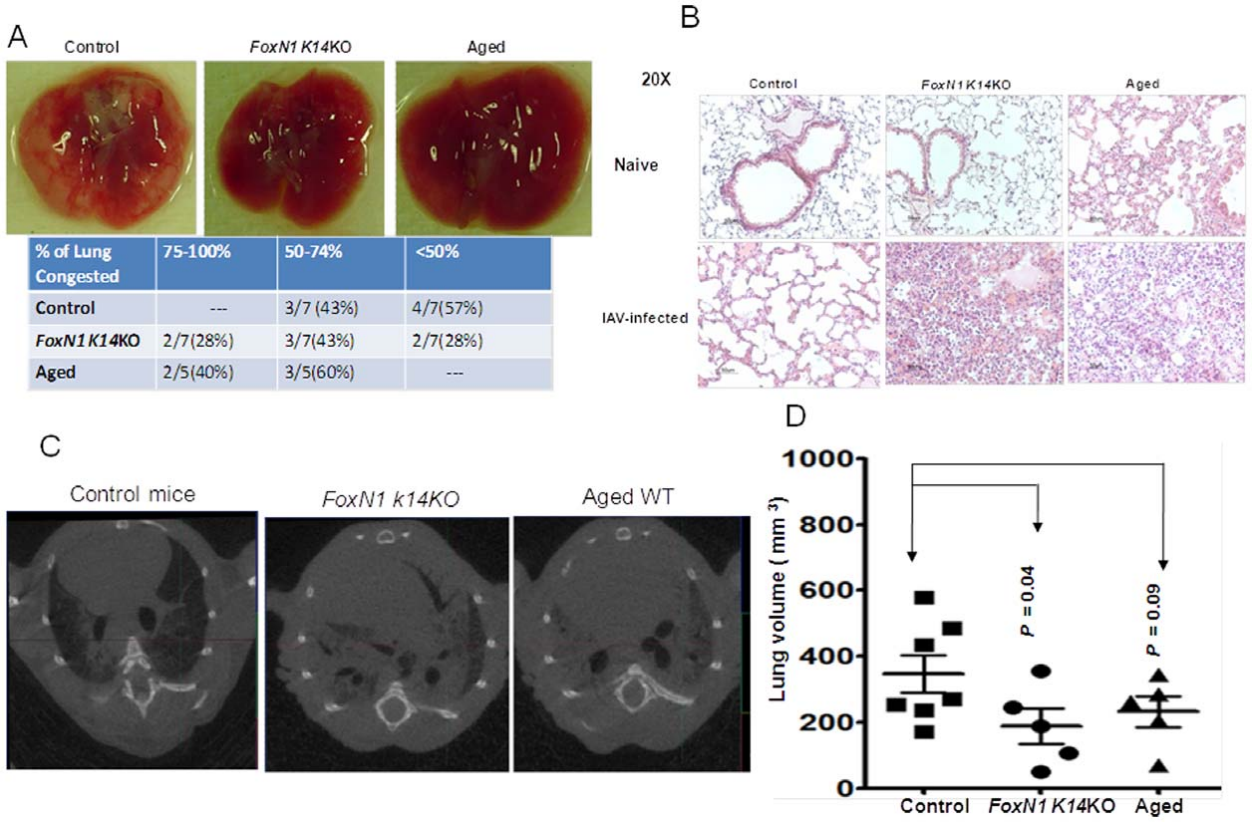
Pathology and anatomic changes in the lungs of *FoxN1 K14KO*, aged and young control mice after infection with influenza virus. (A) Gross anatomy and (B) histopathology of the lungs of *FoxN1 K14KO*, aged and young control mice 10 days post-infection with 0.2 LD50 of PR8. Representative sections (magnification 20×), stained with hematoxylin and eosin, are shown. (C) Mice were infected as in panel A, and were imaged 10 days post infection in a GE eXplore Locus (x-ray tube voltage of 80 kVp, tube **c**urrent of 450 µA). The exposure time was 90 ms per projection and the gantry was rotated over 200 degrees while 200 two-dimensional projections were taken. The images were reconstructed with the eXplore reconstruction utility, and analyzed with Microview software. (D) Ten days post-infection and prior to CT-scanning, lung volumes at peak inspiration in young control, *FoxN1 K14*KO and aged mice were measured. Each dot represents an individual mouse. Means and SEMs are depicted.

We next evaluated the severity of lung injury after influenza infection, using histopathology and micro-CT scanning. On gross examination of the lungs, both *FoxN1 K14*KO and aged mice showed more extensive areas of lung congestion than young control mice (Figure 5A). Microscopic analysis revealed more inflammatory infiltrate and alveolar injury in aged WT mice and in *FoxN1 K14*KO than in control young mice (Figure 5B). Micro-CT scanning of the lungs showed widespread consolidation of the lung in aged WT and *FoxN1 K14*KO mice, but not in control young WT mice (Figure 5C), probably due to the infiltration of inflammatory cells, edema and tissue destruction.

Lung volume is another indicator of lung injury and was measured in all three groups of mice, prior to and after infection with influenza virus PR8. In uninfected animals the lung volumes were comparable in all three groups. Ten days after infection with PR8 virus, mean lung volumes at peak inspiration were 0.35±0.06, 0.19±0.05, and 0.23±0.05 mm^3^ in young control, *FoxN1 K14*KO and aged mice, respectively (Figure 5D).

### *FoxN1 K14*KO mice have higher titers of influenza virus PR8

**Figure 6:**
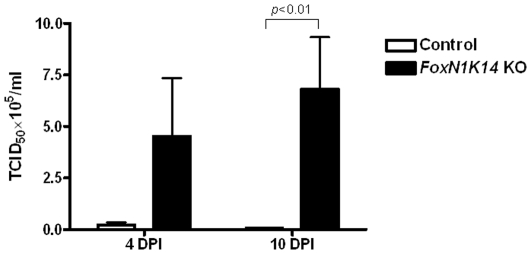
Viral burden. Young control mice (n = 5) and *Foxn1 K14*KO mice (n = 5) were infected with 0.2 LD50 of PR8 virus. Four and 10 days post infection (DPI), lungs were aseptically removed at the bronchi, snap-frozen and kept at −70°C. Lungs were then thawed and homogenized. Ten-fold dilutions of lung homogenates were applied to confluent MDCK cells. After 72 hrs, the viral cytopathic effect was recorded and the median tissue culture infective dose (TCID50) was determined. Means and SEMs of 5 mice/group are shown.

We next measured the influenza virus titers in the lungs after infection with a sublethal dose (0.2 LD50) of PR8 virus. *FoxN1 K14*KO mice had significantly higher titers than young control mice, 4 and 10 days post-infection (Figure 6).

## Discussion

The thymus is the primary source of T cells, and TECs play a central role in creating a microenvironment that permits normal development and release of naïve T-cells to the periphery. The forkhead-class transcription factor, encoded by *FoxN1*, regulates development, differentiation, and function of TECs, both in the prenatal [11], [12] and postnatal thymus [13], [14]. We recently found that deletion of *FoxN1*in K14-expressing TECs resulted in accumulation of thymic cysts, loss of normal three-dimensional thymic architecture and reduced numbers of mature CD4+ and CD8+ T-cells [4]. In the current report, we showed that these mice had reduced numbers of peripheral CD62L+CD44− naïve T-cells. In addition, during influenza infection, these animals had reduced antigen-specific CD8+ T-cell and IgG responses to influenza virus, combined with increased lung injury, weight loss and mortality. These findings paralleled those observed in aged WT mice, providing the first evidence that K14-mediated *FoxN1* deletion causes changes in T-cell function that mimic those in aging during a physiologically relevant response to challenge with an infectious agent.

FoxN1 has long been known to be essential for normal prenatal thymic development [15], and recent work, using a *FoxN1^lacZ^* mutant [13] and a conditional deletion of *FoxN1* [14], indicates that this transcription factor is also required for maintenance of the postnatal thymus. Normal aging is accompanied by thymic involution, reduced thymocyte output and reduced expression of *FoxN1* [16], [17]. Genetically modified mice with progressive loss of *FoxN1* showed accelerated thymic involution, with gross and microanatomic changes that mimicked those of natural aging [17]. Furthermore, intrathymic injection of *FoxN1* cDNA to aged mice increased thymic size and thymocyte numbers [17]. The sum of these findings suggest that loss of FoxN1 contributes causally to the intrathymic changes of aging. However, limited information is available on how changes in *FoxN1* expression affect peripheral T-cell function under physiologic conditions. Aging results in marked reductions in the number of naïve T-cells [18], and this in turn limits the capacity of T-cells to respond to infection, resulting in more severe disease. The current report demonstrates that *FoxN1 K14*KO mice also have fewer naïve T-cells and reduced CD8+ T-cell responses to influenza infection, with increased lung injury, weight loss and mortality. The K14 epithelial cell marker is largely confined to the medullary thymic epithelium and is absent from the cortex [19]. Therefore, our results provide strong evidence that deletion of *FoxN1* in medullary thymic epithelial cells is sufficient to mimic the effects of aging on peripheral T-cell function.

During normal aging of the cell-mediated immune system, the decline in naïve T-cell number is due in part to reduced production of naïve T-cells by the thymus and in part to expansion of peripheral memory T-cells, due to lifelong antigen exposure. Because young *FoxN1 K14*KO mice have not had extensive antigen exposure, the reduced number of naïve T-cells may be due primarily to reduced thymic output, consistent with findings that the naïve T-cell repertoire is comparably reduced in young thymectomized mice and aged mice with intact thyme [18]. On the other hand, loss of FoxN1 also causes inadequate negative selection in the thymus (Guo *et al.* unpublished data), resulting in self-reactive T-cells that are triggered in the periphery, and then express memory markers, as is the case in *FoxN1^Δ/Δ^* mice with a hypomorphic *FoxN1* allele and abnormal TEC development [20].

Aging leads to increased susceptibility to infection, and poor antibody and T-cell responses to vaccines [8], [9], [18], [21]. Protective immunity in influenza is mediated by T- and B-cells, and the immune response to influenza is impaired in the elderly [9]. After infection with influenza virus, the number of influenza-specific CD8+ T cells in the lung and spleen were reduced by approximately 50% in young *FoxN1 K14*KO and aged WT mice, compared to young control mice (Figures 3A and B), indicating that *FoxN1* deletion and aging have similar effects in reducing antigen-specific responses, perhaps because of reduced availability of naïve T-cells. In contrast to findings with CD8+ cells, the frequencies of influenza-specific CD4+ cells were not reduced in aged or *FoxN1 K14*KO mice (Figure 3C), consistent with findings that cytolytic T-cell activity and perforin expression by CD4+ cells are normal in the elderly after influenza vaccination, whereas these functions are impaired in CD8+ cells [22].

The antibody response to influenza vaccination is reduced in the elderly [8], and we found lower H1-specific IgG levels in aged WT and young *FoxN1* K14KO mice. Because *FoxN1* K14KO mice do not have primary B cell defects, this suggests that the capacity of CD4+ T cells to provide help to B cells is reduced in these animals.

The diminished CD8+ T-cell and B-cell responses to influenza in *FoxN1 K14*KO mice were biologically relevant and consistent with results from aged mice [10]. *FoxN1 K14*KO mice showed higher viral burdens, increased lung damage, weight loss and mortality, compared to control young mice. In humans, the elderly are also more susceptible to complications and death from influenza than young adults [9]. Weight loss was more severe and death was more common in aged mice than in *FoxN1 KI4*KO mice (Figure 4), suggesting that defects other than reduced T- and B-cell responses, such as baseline lung function and changes in other cells, such as alveolar macrophages, may contribute to morbidity and mortality due to influenza in aged animals.

In summary, we found that deletion of *FoxN1* in K14-expressing thymic medullary epithelial cells reduces the number of naïve T-cells, decreases the antigen-specific CD8+ T-cell and B-cell response during influenza infection, and increases lung injury, weight loss and mortality. These findings mirror those in aged WT mice, demonstrating that *FoxN1* deletion causes T-cell defects that mimic those in aging during the response to infection.

## Materials and Methods

### Mice

*FoxN1 K14*KO young mice were generated and genotyped, using the *loxP* Cre-recombinase system, as described previously [4]. Young control mice were those in which the *Foxn1* gene was flanked by *loxP* sites, but did not contain K14-driven Cre recombinase, and were therefore physiologically WT mice (http://cre.jax.org/Krt14/Krt14-creNano.html). Aged 20 month-old C57BL/6 WT mice were purchased from the National Institute of Aging. In accordance with NIH guidelines, all protocols involving animals were approved by the Institutional Animal Care and Use Committee at the University of Texas Health Science Center at Tyler.

### Influenza A virus infection

We used a mouse-adapted influenza virus strain, A/Puerto Rico/8/34 (H1N1, PR8, Charles River, North Franklin, CT). Mice were anesthetized with an intraperitoneal injection of a mixture of ketamine and xylazine, and infected intranasally with 50 µl of virus. Following infection, mice were monitored daily for weight loss and mortality.

### Histopathology

Lung tissues from WT and transgenic mice were fixed in 10% neutral buffered formalin and embedded in paraffin. Five µM serial sections were made, stained with hematoxylin and eosin, and evaluated by standard methods under an Olympus DP70 microscope.

### CT scan

Mice were subjected to a non-survival CT scan, 10 days after infection with influenza, using a GE eXplore Locus at an x-ray tube voltage of 80 kVp and a tube current of 450 µA. The exposure time was set to 90 ms per projection and the gantry was rotated over 200 degrees while 200 two-dimensional projections were taken. The images were reconstructed using the eXplore reconstruction utility. Images were analyzed using Microview software.

### Measurement of lung volume

Animals were deeply anesthetized with a mixture of ketamine and xylazine (60 mg/5 mg) ip. The trachea was cannulated using a G20 catheter. Animals were ventilated at a respiratory rate of 150 breaths/min, as recommended by the manufacturer. To acclimate to the Flexivent ventilator, mice were allowed a 5 min resting period before the experiment began [23]. Forced pulmonary maneuvers were used to measure lung volumes.

### Viral quantitation

Madin-Darby Canine Kidney (MDCK; ATCC®: CRL-2936™) cells were cultured overnight in 96-well-plates with complete medium containing MEM, 10% FBS, 0.1 mM non-essential amino acids, and 1.0 mM sodium pyruvate. The next day, lung homogenates were serially diluted 10-fold with MEM and loaded on confluent MDCK cells. After 1 h of absorption, plates were washed with Hanks' buffer and complete medium containing MEM, 0.1% BSA, 0.5 µg/ml TPCK-trypsin, 100 U/ml of penicillin and 100 µg/ml streptomycin was added and incubated at 37°C and 5% CO_2_. Seventy-two h later, cytopathogenic changes were quantified under a microscope, and the 50% tissue culture infective dose (TCID_50_) was calculated by the Spearman-Karber formula.

### Flow cytometry

Cells were isolated from the lungs and spleens and stained with antibodies to cell surface markers: murine CD3 (Mac-1a), CD3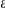 (145-2C11), CD4 (L3T4), CD8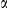 (53-6.7) (all from eBioscience, San Diego, CA). For pentamer staining, cells were incubated with MHC class I (H-2Db) pentamers specific for influenza virus NP_366–374_ (ASNENMETM, ProImmune Ltd., Oxford, United Kingdom) for 10 min at room temperature, followed by incubation with anti-CD8-FITC. To measure proliferation, spleen T cells were negative selected with the Pan T cell isolation kit (Miltenyi Biotec, Auburn, CA) and incubated with 2 µM CFSE. Cells were placed in 24-well plates pre-coated with anti-CD3 and anti-CD28, at 37°C. At different time points, cells were harvested, stained with cell surface markers and analyzed by flow cytometry, using a BD FACS Calibur (BD, San Jose, CA) and FlowJo software (TreeStar, Inc., Ashland, OR).

### ELISPOT assay

To determine the frequency of influenza-specific IFN-γ-producing CD4+ T cells, ELISPOT plates were coated overnight with 10 µg/ml anti-mouse IFN-γ at 4°C. CD4+ T cells were positively selected from splenocytes by CD4 microbeads (Miltenyi Biotec). Antigen-presenting cells were prepared by incubating naïve splenocytes with medium or PR8 virus at a multiplicity of infection of 1 for 3 h at 37°C. CD4+ T cells and antigen-presenting cells were cultured at a 5∶1 ratio in the pre-coated ELISPOT plates in duplicate and incubated at 37°C overnight. Plates were developed using the detection antibody, R4-6A2-Biotin (Mabtech, Cincinnati, OH), followed by streptavidin alkaline phosphatase (Mabtech) and BCIP-NBT substrate solution (BD Bioscience). Plates were rinsed with tap water and air dried, and spots were counted with a stereomicroscope (Olympus SZ2-LGB).

### ELISA to measure influenza-specific antibodies

Influenza-specific IgA and IgG1/IgG2a were measured by ELISA in lung homogenates and serum, respectively. Briefly, 96-well immunoassay plates were coated overnight with 2 µg/ml PR8 virus at 4°C. After blocking, lung homogenates or serum were added to each well in serial dilutions and incubated overnight at 4°C. Bound antibodies were detected by adding biotin-conjugated anti-mouse IgA (1∶1000, BD Bioscience) followed by streptavidin-HRP or HRP-conjugated IgG1(1∶4000) or IgG2a (1∶1000) (Southern Biotech, Birmingham, AL) at room temperature. Plates were developed with TMB substrate solution, stopped with 500 mM HCl, and OD values were measured at 450 nm.

### Statistics

Comparisons between groups were calculated by an unpaired Student's *t* test; *p*<0.05 was considered statistically significant. All measures of variance are presented as SEMs.
